# Rise of the machines: The future may be here for food allergy diagnostics

**DOI:** 10.1016/j.jacig.2024.100301

**Published:** 2024-07-10

**Authors:** Edwin H. Kim

**Affiliations:** From the Department of Pediatrics, University of North Carolina School of Medicine, University of North Carolina at Chapel Hill, Chapel Hill, NC

In response to the well-documented rise in food allergy prevalence, the field of food allergy has seen a rapid growth in research. Pivotal discoveries in the field over the past 20 years are highlighted by the LEAP study,^1^ which has ushered in the paradigm-shifting approach of early introduction of peanut to prevent allergy, and the PALISADE study of oral immunotherapy,^2^ which has begun to shift the mindset of food allergy management from passive avoidance to proactive desensitization. Following on the heels of these studies, numerous other novel efforts toward both the prevention and treatment of food allergy are ongoing and likely to significantly change how food allergy is practiced in the future. On the other hand, one area of food allergy that has not seen the same level of change has been the diagnosis of food allergy. Although some advances (such as the advent of component-resolved testing, epitope mapping, and basophil activation testing) have been made, clinical practice has remained reliant on skin prick testing (SPT) and/or allergen specific IgE testing, with oral food challenge (OFC) still held as the “criterion standard.”^3^ Unfortunately, SPT and IgE testing are prone to false-positive results, in particular, when used in broad panel screening for food allergy. OFC is more definitive; however, it involves the risk of anaphylaxis, is time-consuming, and is staff-intensive. As a result, only a minority of allergy clinics offer food challenges, significantly limiting access to the procedure and exacerbating concerns about a lack of diversity in the diagnosis of food allergy.

With a growing appreciation for the limitations brought on by a reliance on OFCs, initiatives to “replace” OFC have developed; these initiatives include the Food Allergy Research and Education Innovation Award Diagnostic Challenge and the US Food and Drug Administration Biomarker–Driven Drug Development for Allergic Diseases and Asthma public workshop. These efforts bring hope for the development of biomarkers that can greatly improve on our current testing modalities. Although the arrival of the perfect replacement for OFC would be universally embraced by allergists, it may be more realistic to think that these technologies may reduce our need for diagnostic OFCs rather than fully replace them. The ability to reduce the large “gray zone” that exists with regard to our current diagnostics and reserve OFCs for the truly equivocal cases would still provide a dramatic improvement compared with today’s process, which leaves many patients with unclear and possibly incorrect diagnoses. In addition, rather than the current push for more clinics to offer OFCs, many of which may not be adequately set up to perform challenges, reducing the need for OFCs would better fit with the current system of OFC referral centers.

In their article “Prediction of pediatric peanut oral food challenge outcomes using machine learning,” Gryak et al^4^ make the case that the solution may not need to be a new technology or modality but may instead already be available to us. They make the point that the logistic regression (LR) model of Dunn-Galvin considering basic biomarkers such as SPT and allergen-specific IgE had promising results but was heavily reliant on the severity of the prior reaction, thus limiting its generalizability.^5^ Gryak et al^4^ build off their previously described machine learning algorithm^6^ to train and assess several predictive models using the well-characterized publicly available data set from the LEAP prevention study.^1^ These were then validated against the publicly available IMPACT peanut oral immunotherapy data set^7^ and the OFC data set from the University of Michigan repository. The included variables were expanded to include peanut component testing as well as SPT flare as additional variables commonly available in clinical practice. A sensitivity of 0.986, specificity of 1.0, and area under the curve of 0.993 were achieved by the best-performing machine learning algorithm on the LEAP data set. For comparison, multivariable LR analysis provided a sensitivity of 0.942, specificity of 0.778, and area under the curve of 0.860. Machine learning similarly outperformed LR in the IMPACT and Michigan replication data sets. Interestingly, when the goal of analysis was to identify the most impactful variables in the models, flare size, which is often deemphasized in clinical practice, rose to the top.

The findings of Gryak et al^4^ are limited by their study’s focus on a single food, in this case peanut, and the inclusion of patients who were under consideration for clinical trials and thus had a higher risk of allergy. However, the results support the idea that machine learning approaches can take large amounts of data into consideration and improve on standard statistical approaches such as LR. Validation of the model across the additional unrelated cohorts was reassuring for the generalizability of the approach. As is inherent in machine learning approaches, the continuous addition of data sets to the model would be expected to improve on its performance. Successful extrapolation of the approach to other foods would be dependent on the availability of data sets to train the algorithms but would be expected to similarly improve on individual variables and LR of multiple variables.

A 100% accurate biomarker to replace OFC remains the dream for food allergy; however, the technologies currently in development do not appear close to achieving this goal. Even if there were a promising candidate, the rigorous process for US Food and Drug Administration approval of such a technology would likely put the technology years away from clinical practice. This may be a case of perfection being the enemy of good. The machine learning approach showcased by Gryak et al^4^ takes commonly available clinical variables and provides a result that may not be perfect but could be very good by accurately diagnosing a larger proportion of patients and greatly reducing the number actually needing OFCs. A coordinated effort across clinics and research studies to uniformly collect OFC data, including SPT flare, could rapidly improve these models and hasten their introduction into clinical practice. The machines have risen, and with them, the future of food allergy diagnostics may be here.

## Disclosure statement

Disclosure of potential conflict of interest: E. H. Kim reports advisory board membership with ALK, Kenota Health, and Ukko Inc; consultancy with Allakos, Cellergy Pharma, DBV Technologies, Genentech, Hanimmune Therapeutics, Novartis, Phylaxis, and Revolo Biotherapeutics; and research grants to his university from the National Institutes of Health and Food Allergy Research and Education (FARE).
